# Impact of News Overload on Social Media News Curation: Mediating Role of News Avoidance

**DOI:** 10.3389/fpsyg.2022.865246

**Published:** 2022-04-12

**Authors:** Xiao Zhang, Shamim Akhter, Abdelmohsen A. Nassani, Mohamed Haffar

**Affiliations:** ^1^Sichuan Radio and Television Station, Chengdu, China; ^2^School of Languages, Civilisation and Philosophy, Universiti Utara Malaysia, Kedah Darul Aman, Malaysia; ^3^Department of Management, College of Business Administration, King Saud University, Riyadh, Saudi Arabia; ^4^Department of Management, Birmingham Business School, University of Birmingham, Birmingham, United Kingdom

**Keywords:** news overload, quantitative approach, media strategies, news curation, news avoid

## Abstract

In this global village, easy access to news has resulted in many changes in the preferences and patterns of people for accessing news. Therefore, the present study has attempted to investigate the effects of news relevance, perceived quality, and news overloading on people’s news curation preferences. This study has also examined the mediating role of news avoidance between the news relevance, perceived quality, and news overloading on the news curation. A quantitative technique has been employed to check the relationships proposed in the study. A sample size of 217 has been analyzed to check these hypotheses. The findings of the study revealed that news relevance has a negative impact while news overload positively and significantly impact the news curation. However, the news avoidance only mediated the relationship of news quality and news curation. Theoretically, the study has contributed to the literature of journalism and social media by finding that relevance of news has a negative significant impact on the news curation behavior of people, such that if they do not find the news relevant, they happily curate it. Practically, the study implies that the people are more prone to the quality of news rather than quantity; therefore, it is important for the news agencies to ensure that they produce and deliver the quality based news considering the accuracy to penetrate the Theoretic lass level.

## Introduction

A stream of news from an increasing number of sources and platforms is overburdening news consumers, leading to exhaustion as well as indifference to the news (Tandon et al., 2022). Individuals are bombarded with an overwhelming volume of information as the line between news creation and consumption blur and more channels and technologies become accessible for news consumption (Peng, 2022). Furthermore, because of the increased use of mobile devices, news is transmitted more swiftly to a larger number of people than ever before. The problem of news overload has been increased by constant news updates on social media and smartphone applications. The news element of information overload is the subject of this study. Previous research on news overload has largely focused on the levels of news overload as well as the human traits that influence news overload (Bachmann et al., 2021).

The word “information overload” is commonly used to describe obtaining an excessive amount of data. When humans are exposed to even more information than their knowledge processing capabilities can handle, they experience information overload (Matthes et al., 2020). Previous research has shown that massive amounts of data are disruptive and have a detrimental influence on productivity and decision making. Because the pace at which information is produced and disseminated has increased dramatically as a result of ICT growth, these negative impacts become exaggerated (Lee et al., 2020). An excessive amount of information, unintentional expansions of social networking, and quick changes in the technological aspects of social networking services are all examples of new sorts of overload introduced by the growth of social media (Tugtekin et al., 2020). Information fatigue is linked to the energy consumption needed by these task loads.

According to other research, the majority of users who temporarily leave Facebook are because the site is seen as a strain on cognitive resources (Islam et al., 2022). Because the volume of news and accessible channels for news consumption has significantly risen, the Internet and social media have prompted study into rising perceptions of information load in a media environment. Women were more likely than males to feel overburdened, and older people were more inclined to agree (Hasan and Bao, 2020). Females have a harder time digesting news material, and older persons may have worse cognitive processing efficiency, making both categories more susceptible to news overload. The study also found that gender (i.e., being female) was connected to perceived news overload.

People can deal with information overload by avoiding and neglecting the overwhelming amount of data available (Soroya et al., 2021). When presented with an overload, people use two different techniques to deal with it. One option is to manage the data by lowering the quantity of data processed or by employing more efficient processing strategies such as organization, priority, and management procedures. The second option is to permanently shut down by rejecting or avoiding information (Kearney and Childs, 2021). People would eventually experience news overload as the volume of news grows, causing them to shuttered intellectually and reject the importance of news intake in forming a worldview as well as conducting a social debate (Avotra et al., 2021). People who are unable to effectively control their overload are much more prefer to avoid obtaining further information.

The excess of knowledge itself is not always seen as a bad thing because it gives information consumers extra capabilities (García-de-Frutos and Estrella-Ramón, 2021). Nevertheless, when various media rebroadcast the same news with slightly altered versions, the supply of information is expanded (Zhang M. et al., 2022). People who are experiencing overload but still want to receive news items must control their overload in this scenario (Tandon et al., 2022). Depending on how information is handled, there are two types of strategies for dealing with overload.

The survey did not represent several aspects that might also influence these practices and should be considered for a coherent theoretical model that situates personal practices of news curation in the existing literature on selective exposure and incidental news exposure. Nonetheless, the previous research only measured news overload in terms of quantity as the antecedent of news curation however did not measure the perceived quality and relevance of news. The present study has incorporated these variables to fill the gap in the literature. The link between perceived news overload, news relevancy, and news curation is investigated in this study.

In this study, Chapter 1 is representing introduction of the study. Chapter 2 is based on theoretical and hypothesis support, Literature review and hypothesis. Chapter 3 is based on Methodology including demographic details, measurement and statistical tools. Chapter 4 is based on results. Chapter 5 showed the discussion, limitation, and future recommendation. Chapter 6 is based on conclusion, respectively.

## Theoretical and Hypothesis Support

The concept of news overload and news avoidance are drawn from cognitive-load theory (Sweller, 1988). According to cognitive-load theory, people’s brains only have so much capacity, that they must control how much information they take in Paas et al. (2003). When technology is used beyond its optimal level, it can have negative consequences (Karr-Wisniewski and Lu, 2010). News information overload seems to be a type of cognitive barrier that obstructs or restricts the user’s ability to gather information and frustrates them (Savolainen et al., 2018). Individuals who think that news overload through social networks may use this avoidance method (Song et al., 2016). Individuals might try to avoid as much as possible the overabundance of news (Savolainen, 2007). People increasingly sense news overload as the quantity of news exposure grows, which might cause people to shutter cognitively, dismiss the importance of news intake, or put less effort into getting news. Individuals have a propensity to shield themselves against information overload (Savolainen, 2007). News curation is a cognitive knowledge procedure that provides connotative and/or associative significance to incoming information. The first stage in curation is proper understanding and interpretation of news through cognitive reflection (Park and Kaye, 2018). News curation, on the other hand, takes extra work in terms of studying, evaluating, and understanding about the topics in order to help others have a better understanding of the political arena. The cognitive tuning theory by Zajonc (1960) proposes that as individuals prepare to transfer knowledge, the content should be organized well, understood, and even sometimes memorized, since they sense a need to completely comprehend what they are providing (Cacciatore et al., 2018). This theory provided basis for news curation and we utilized in current research as a dependent variable. Similarly relevance of news is drawn from relevance theory (Foster-Cohen, 2000; Chapman, 2005). Relevance is a difficult concept to define, in least since our job lies at the crossroads of several areas, each of which defines it uniquely. Relevance theory is most likely the most influential within pragmatics. Relevancy is a trade-off between acquiring the most recent information while exerting the least amount of effort. Importantly, in this viewpoint, relevance is always relative rather than absolute: people will consider what is most important at any given time.

Relevance theory describes a relevant input as one that “makes a meaningful contribution to the person’s perception of the world,” based on Grice’s succinct guideline “be relevant” (Grice, 1975). Relevancy is an exchange between getting the most recent information while putting in the least amount of effort. Importantly, in this perspective, relevance is always relative rather than absolute: people will consider what is most essential at any particular time (Barchas-Lichtenstein et al., 2021). Meanwhile, relevance has been largely overlooked in the language research on news values. Bednarek adds in a review of linguists’ handling of news values that those who included relevance often have supplied ambiguous interpretations, and Bednarek primarily encompasses this element under the criterion of impact (Bednarek, 2016; Huan, 2018). Therefore, based on this relevance theory, we proposed relevance of news as an element of impacting factor toward news curation in this research.

### Relevance of News Has an Effect on News Curation

Relevance of news is a cloud-based information analytics software provider that provides real-time representations of data linkages from various sources (Antomarioni et al., 2022). Current audience research acknowledges the media-saturated character of everyday life, but the function of journalism for individuals living in this digitalized world is less obvious (Vanden Abeele et al., 2022). The argument for integrating two contrasting analytical perspectives in cultural audience research that depend on the framework of practice theory in order to create a better understanding of the role of journalism and news in everyday life. Reporters may structure news to emphasize their relevance to a certain readership, and readers and viewers may develop extra deep ties that aren’t explicitly stated in the article or arise just through conversation with each other. Prior study has looked into the language skills used by reporters to express the importance of news and news principles in general.

Vidali (2010), for instance, noted mainstream news viewers’ behavior *via* pronouns, participatory frameworks, and socially constructed ideologies of sincerity, whereas (Molek-Kozakowska, 2016) pointed out that “events are not always intrinsically newsworthy, but can be crafted as newsworthy with particular application of pictures and linguistic devices.” There has been little research into how readers and viewers build relevance discursively, but there is reason to suppose they vary from journalism (Armstrong et al., 2015; Cunningham et al., 2016). Busse and Gray’s (2011) report concentrated on news talk, which is defined as “the unstructured and often highly productive manner that news articles are shared among individuals, and interpretations are constructed that may or may not have any relevance with initial purpose of the reporter who wrote the news”. Social media platforms such as Facebook, Twitter, and Instagram have become an essential aspect of online news dissemination and consumption during the last two decades.

In response to feedback for theoretical creativity in the aftermath of this development (Paceley et al., 2022). To conceptualize social media news exposure, use the notion of “filtered flows.” They argue that on social networking sites, “curation” processes—the creation, selection, filtering, analysis, or framing of content – are carried out by a wide range of actors, including not only traditional newsmakers and gatekeepers like journalists, but also politicians, online social contacts, algorithms, and individual users (Zhang H. et al., 2022). According to a multiple years based research conducted in Finland, readers and viewers recognize the importance of journalism in their everyday lives, particularly in relation to their social networking (Heikkilä et al., 2010; Heikkilä and Ahva, 2015). There has been no research, which previously assessed the impact of news relevance on news curation but this literature proved a scope for future research therefore, we hypothesized the following.

***H*****_1_*:*** *Relevance of news has an effect on news curation.*

### Perceived Quality of News Has an Effect on News Curation

The concept and perception of news quality have shifted for viewers as a result of digital media’s transformation of not just news forms but also producing and dissemination methods (Bengtsson and Johansson, 2020). Younger viewers, for example, prefer to play a key role in current news habits, as they distribute news and create debates, as well as participate in them (Burkey, 2019). As a result of modernization, viewers’ perceptions of quality and the objectives for which they obtain news are likely to have shifted. Quality perceptions inside the industry of reporting as well as from the perspective of users are not always in sync (Edgerly, 2021). As a result, studies on broadcast quality differentiates between journalistic excellence and users’ perceptions of quality (Bachmann et al., 2021). Rather than viewing news as something that “should be known” and using quality standards like diversity or relevance, young viewers see news as something that is helpful, engaging, and entertaining to learn about (Galan et al., 2019).

According to a recent study, younger folks evaluate news based on its quality, truthfulness, and capacity to facilitate sociability (Swart, 2021). This encompasses characteristics of their daily life and social interactions (sociability), as well as traditional journalistic standards (accuracy and truthfulness) in their news quality ratings. Further it has been concluded that young consumers are educated and enjoy news that fulfills certain quality requirements. According to a Finnish research, young individuals want greater inquiry, confirmation of media content, and trustworthy sources (Manninen, 2020). Since younger folks value news in their daily lives, but do not always absorb it actively, making them “a population that is of tremendous interest to news producers throughout the world, and one which they are finding more difficult to reach” (Schwaiger et al., 2022). As per this suggested literature on perceived quality of news among people, we assumed that such perceived quality of news could have an impact of news curation as well therefore, we proposed the following hypothesis.

***H*****_2_*:*** *Perceived quality of news has an effect on news curation.*

### News Overload Has an Effect on News Curation

The terminology “information overload” is commonly used to describe obtaining too much information (Wang et al., 2022). When participants were exposed to more information than their information processing capabilities to handle, they experience information overload (Apuke et al., 2022). Recent research has discovered that large quantities of information may be disruptive and also have a strong impudence on productivity as well as decision making (Ramzan et al., 2022). These negative implications are further magnified since the efficiency of producing and disseminating information has increased dramatically with the advancement of ICT (Liu et al., 2022). Apart from analyzing demographics to determine degrees of felt news overload, watching and reading news *via* digital platforms such as computers, e-readers, plus Facebook exhibited a positive connection with perceived news overload (Lee et al., 2022). However, traditional news sources such as newspapers, television, and magazines did not result in sensations of news overload.

In other words, when consumers consumed news *via* digital devices, they felt a higher news overload (Goyanes et al., 2021). The quantity of information is not always seen negatively because it presents information searchers with more options (Lee and Hong, 2021). However, the amount of information is enhanced since many news organizations republish the same or slightly modified versions of the very same news (Mourão and Robertson, 2019). People who sense overload but wish to continue viewing news items must control their overload in this case (Tunney et al., 2021). There are two sorts of strategies for dealing with information overload, based on how the information is handled. To begin, the filtering method is predicated on the need to concentrate on the most valuable information by carefully removing worthless data from sources (Antomarioni et al., 2022). Secondly, the escape strategy is largely motivated by emotive considerations stressing the personal desire to protect oneself from information overload.

News is currently filtered through several channels and delivered to customers *via* a range of platforms known as news curation services (Martin, 2021). Yahoo News Digest, for example, is a smartphone app that refreshes twice a day with the top eight news articles from across the world. The Circa News app gathers information from a number of sources as well as repackages it into narrative strands (Coddington, 2020). Flipboard is a visually rich news aggregator for phones and tablets that collects material from a range of sources and organizes it into themed collections. After then, readers can track collections, themes, or publications (Vanden Abeele et al., 2022). Social media, as well as various news channels, may serve as news curating services. Previously, there has been no study conducted to evaluate the impact of news overload on news curation but there remained a gap to address this relationship so, we developed the following hypothesis in this regard.

***H*****_3_*:*** *News overload has an effect on news curation.*

### Mediating Relationship of News Avoidance

A flow of news from an increasing number of sources and platforms is overburdening news consumers, leading to exhaustion and apathy to the news (Toff and Palmer, 2019). Individuals are confronted with an unprecedented volume of information as the line between news creation and consumption blurs and more channels and technologies become accessible for news consumption. When there is an abundance of news, it acts like noise (García-de-Frutos and Estrella-Ramón, 2021). Anxiety, boredom, excessive redundancy (receiving meaningless information repeatedly), and distraction are common side effects of information overload (Lee and Hong, 2021). Such exhaustion is common in the workplace, and the name “information fatigue syndrome” was developed to describe it (Thomas, 1998). Extreme stress and emotions of powerlessness, pessimism, and pressure are among the symptoms. We believe that news consumers who experience overload as a result of too much news will suffer from tiredness.

In a media world where a wealth of news is available for 24 h a day, it is a contradiction that an increasing number of people are turning their backs on the news, resulting in widening inequities in political awareness and involvement (Van Aelst et al., 2017). Conversely, some new research of information dissemination have discovered a large population of individuals who read an watch no or just a little bit of news, referring them as minimalists, news avoiders, intermittent or non-users (Wolfsfeld et al., 2015; Mosca and Quaranta, 2016; Strömbäck et al., 2018). Considering the increasing interest in the topic, academics are still divided on the breadth of news avoidance. One South Korean research related to national representative samples labeled approximately 73% of participants as news avoiders (Lee and Yang, 2014), whereas Dutch researchers showed just 11% news avoiders (Trilling and Schoenbach, 2012) and a Swedish study found 15% news avoiders (Strömbäck et al., 2013). Several other research shows percentages that fall somewhere in the middle of these two extremes (Bos et al., 2016).

This significant disparity is most likely due to differences in the conception and operational definitions of news avoidance, at least to some extent. Because of these discrepancies, determining the level of news avoidance overall, let alone comparing news avoidance throughout various media platforms, is challenging (Skovsgaard and Andersen, 2020). A lot of research has undergone in past to examine different roles of different factors contributing toward news avoidance including quality of news, relevance of news, efficacy of news, and new overload, etc., but no prior research has ever found mediating role of news avoidance in any context. It could lead to news curation as suggested in our research context, so we proposed that direct relationships of perceived news quality, relevance of news, and news overload with news curation could be mediated through news avoidance as well. In this regard we proposed the following.

***H*****_4_*:*** *News avoidance mediates the relationship between relevance of news and news curation.*

***H*****_5_*:*** *News avoidance mediates the relationship between perceived quality of news and news curation.*

***H*** **_6_** *: News avoidance mediates the relationship between news overload and news curation.*

## Methodology

The present study has quantitatively analyzed the effects of independent variables (i.e., relevance of news, perceived quality and the news overload) on the dependent variable (news curation) and thus followed a deductive approach. Furthermore, the population targeted in this study was the adults using social media for news. The sample was selected based on the convenience sampling to make data collection easier and cost-effective (Avotra et al., 2021). The unit of analysis in this study is the individuals. The data in this study was collected through self-administered survey to make sure the respondents did not face any difficulty. The questionnaire used in the survey had been adapted from the previous studies. The usable questionnaires obtained from the survey were 217.

### Statistical Tool

**FIGURE 1:**
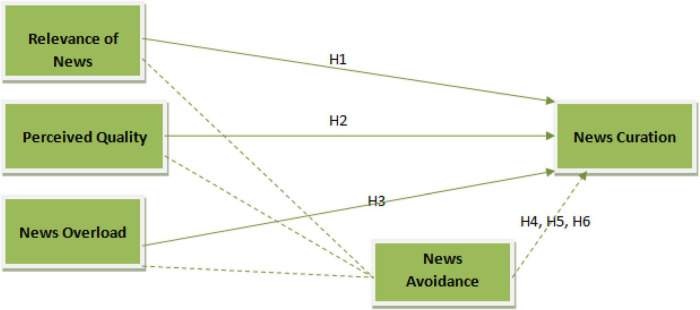
Conceptual framework.

The software used for the analysis of data and hypotheses testing is Smart PLS. This analyses the data through structural equation modeling giving a detailed results for the statistical tests run (Khan et al., 2020). It analyses the data in two stages. The first stage is measurement model that checks the validity and reliability of the data while the second stage of structural model tests the hypotheses developed in the study. Figure 1 shows conceptual framework.

### Measurement

The questionnaire used in this study for collecting the data has been adapted from the past studies. It was curated on a five-point Likert scale giving the options to the respondents from “strongly disagree” to “strongly agree.” There are five variables in the framework designed. The scale for the first independent variable of the relevance of news consisted of three items and it was adapted from Bachmann et al. (2021). The other independent variable of perceived quality has been adapted from Prochazka and Schweiger (2019) and it consisted of four items. The third independent variable of the study, news overload, had been adapted from Chen and Masullo Chen (2019) consisting of six items. The mediating variable of news avoidance had been adapted from Pentina and Tarafdar (2014) consisting of six items. The only dependent variable of the study, news curation, had been adapted from Song et al. (2017) which also comprised of three items.

### Demographic Details

The results of the demographics of the respondents showed that men were dominating in staying updated with the news showing 80% participation by men while the highest number of participants belonged to the age above 50 years followed by age 31–39 and 41–50 years. Furthermore, the highest frequency was found for the respondents having bachelors degree (49.76%) followed by masters. The results for demographic details are given in Table 1.

**TABLE 1:** Demographics analysis.

Demographics	Frequency	Percentage
**Gender**		
Male	172	79.26%
Female	45	20.73%
**Age (years)**		
20 – 30	19	8.75%
31 – 40	73	33.64%
41 – 50	39	17.97%
Above 50	86	39.63%
**Education**		
Bachelors	108	49.76%
Masters	64	29.49%
Ph.D. and others	45	20.73%

*N = 217.*

## Data Analysis and Results

The data in this study has been analyzed using the two-stage approach of structural equation modeling (SEM). The first stage is measurement model, which gives the results of Cronbach alpha, composite reliability along with average variance extracted, Fornell and Larcker criteria and HTMT ratio. Although the other stage of structural model gives the *t*-statistic and *p*-values to check the hypotheses.

### Measurement Model

**FIGURE 2:**
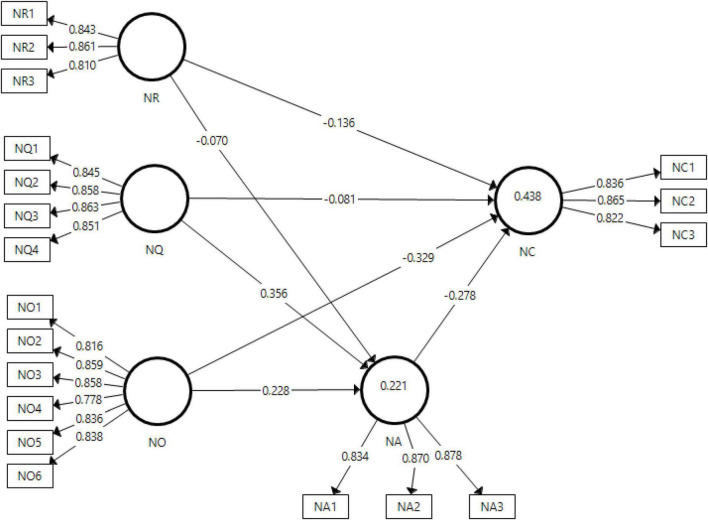
Output of measurement model. NA, news avoidance, NC, news curation, NO, news overload, NQ, perceived quality, NR, relevance of news.

The output of measurement model has been given in Figure 2. It shows the percent contribution of each independent variable in its respective dependent variable.

The measurement model generates the factor loading for each item giving an understanding of the item about it measuring its corresponding variable. According to Shukla and Srivastava (2016) the minimum acceptable value for factor loading of an item is 0.7. The values obtained in this study for factor loadings are all above 0.7 thus meeting the acceptance criteria. Moreover, the acceptability of Cronbach alpha and composite reliability is ensured if they show a value above 0.7 (Sharma, 2020), and the results of the present study met this criteria. Furthermore, the average variance extracted for all the variables was above 0.5 which is the minimum threshold (Zampetakis et al., 2009). The variance inflation factor (VIF) has also been checked in this study to check the collinearity of data. According to Hasan and Bao (2020), the values obtained for VIF should be below 5 to ensure the absence of any issue related to collinearity of data. The values obtained for present study for VIF have also been below 5 and hence meeting the criteria of acceptance regarding collinearity. All these results have been presented in Table 2.

**TABLE 2:** Measurement model.

Variables	Factor Loadings		VIF	Cronbach Alpha	Composite Reliability	AVE
News avoidance	NA1	0.834	1.690			
	NA2	0.870	2.033	**0.826**	**0.896**	**0.742**
	NA3	0.878	2.002			
News curation	NC1	0.836	1.621			
	NC2	0.865	1.848	**0.793**	**0.879**	**0.708**
	NC3	0.822	1.635			
News overload	NO1	0.816	2.541			
	NO2	0.859	3.732			
	NO3	0.858	2.718	**0.911**	**0.931**	**0.691**
	NO4	0.778	2.021			
	NO5	0.836	2.711			
	NO6	0.838	3.383			
Perceived quality	NQ1	0.845	2.000			
	NQ2	0.858	2.337			
	NQ3	0.863	2.244	**0.877**	**0.915**	**0.730**
	NQ4	0.851	2.168			
Relevance of news	NR1	0.793	1.472			
	NR2	0.866	1.752	**0.767**	**0.866**	**0.683**
	NR3	0.818	1.567			

*Bold values indicate that the results for corresponding statistics for the whole variable, not the items.*

To further validate the questionnaire, the study has employed the HTMT ratio test and Fornell and Larcker criteria to guarantee that the variables are distinct from one another. The discriminant validity measured through HTMT ratio should have values not exceeding 0.9 (Franke and Sarstedt, 2019). The obtained values for this test meet the criteria of variables to be distinct and valid. The results of HTMT ratio are shown in Table 3. The second test for the validity of data use in literature is Fornell and Larcker criteria (Henseler et al., 2015). For the results of this test to be valid should show the highest value in diagonal at the top in each variable column. The present study meets this criterion of validity as well. The results can be seen in Table 3.

**TABLE 3:** Discriminant validity (HTMT ratio).

HTMT ratio							Fornell and Larcker criteria				
	NA	NC	NO	NQ	NR		NA	NC	NO	NQ	NR
NA						**NA**	**0.861**				
NC	0.602					**NC**	−0.488	**0.841**			
NO	0.440	0.677				**NO**	0.386	−0.581	**0.831**		
NQ	0.515	0.589	0.649			**NQ**	0.440	−0.493	0.584	**0.854**	
NR	0.508	0.852	0.822	0.774		**NR**	0.405	−0.665	0.687	0.636	**0.826**

*NA, news avoidance, NC, news curation, NO, news overload, NQ, perceived quality, and NR, relevance of news.*

*Bold values indicate that the results are significant showing the highest value of each column at the top.*

R-square also known as coefficient of determination has been used in this study to measure the fitness of model, i.e., how well the values fit the regression line. For present study, the dependent variable of news curation shows 43.8% regression fit while news avoidance shows 22.1%.

### Structural Model

**FIGURE 3:**
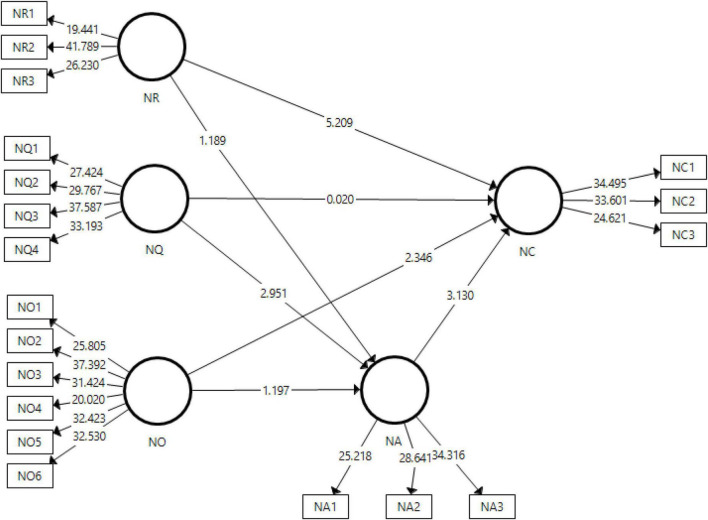
Output of structural model bootstrapping. NA, news avoidance, NC, news curation, NO, news overload, NQ, perceived quality, NR, relevance of news.

Figure 3 of the study shows the output for structural model that is generates the *t*-statistics, β values, and *p*-values that are used to check the proposed hypotheses.

Results for the hypotheses testing have been given in Table 4 indicating both direct and indirect effects checked in the study. The *p*-values should be less than 0.05 in order to accept the proposed hypothesis otherwise it gets rejected (Andrade, 2019). It has further checked the β values, which show the effect of independent variables on the dependent variable (Khan et al., 2021). Furthermore, the inner VIF values have also been reported that should be less than 5 to make sure that collinearity among the variables does not exist (Hasan and Bao, 2020).

**TABLE 4:** Hypotheses testing.

Paths	H	B	Inner VIF	*T*-statistics	*p*-value	Results
**Direct effects**						
NR → NC	H_1_	−0.442	2.278	4.475	0.000*	*Accepted*
NQ → NC	H_2_	0.002	1.899	0.020	0.984	Rejected
NO →NC	H_3_	−0.186	2.056	2.346	0.026*	*Accepted*
**Indirect effects**						
NR→ NA → NC	H_4_	−0.030		1.016	0.324	Rejected
NQ → NA → NC	H_5_	−0.066		2.221	0.027*	*Accepted*
NO → NA → NC	H_6_	−0.030		0.993	0.321	Rejected

N = 217, p* < 0.05, H, hypothesis, NA, news avoidance, NC, news curation, NO, news overload, NQ, perceived quality, NR, relevance of news.

Findings of the study show that six hypotheses had been proposed, three about the direct effects and three indirect through the mediation of news avoidance. Results for the hypotheses testing reveal a significant effect of relevance of news on news curation having *t*-stats = 4.475 and *p* = 0.000, thus accepting the first hypothesis of the study. Regarding the second hypothesis, perceived quality having an effect on news curation has been rejected showing no major. The third hypothesis of the study related to the effect of news overload on news curation has been accepted showing a negative impact (β = −0.186) having *t*-stats = 2.346 and *p* = 0.026. With respect to mediation of news avoidance, no significant effect has been found on the relationships of firstly, relevance of news with news curation and secondly of news overload on news curation thus rejecting the H_4_ and H_5_. However, the mediation of news avoidance has been found significant between perceived quality and news curation having *t*-stats = 2.34 and *p* = 0.7.

## Discussion

This research was designed to evaluate different contributors for news curation. Three direct and three indirect relationships were studied in this research. News curation is a cognitive knowledge procedure that provides incoming information connotative and/or associative significance. Proper understanding of a news and interpreting it by cognitive reflection is the first stage in curation (Park and Kaye, 2018). News curation, on the other hand, takes extra work in terms of studying, evaluating, and understanding about the topics in order to help others have a better understanding of the current affairs. For evaluating news curation in this regard, impact of news relevance was estimated on news curation. The results revealed that relevance of news had significant contribution among the respondents toward news curation. The reason for such a significant relationship lies in the fact that if social media users get relevant news from any source, they are seeking for; they tend to share it with others immediately through social media.

There is very little research as to how readers and viewers build relevance discursively, however there is reason to suppose they vary from journalistic point of view (Armstrong et al., 2015; Cunningham et al., 2016). The other direct relationship of perceived quality of news toward news curation did not indicate any significant contribution toward news curation. The possible reason behind such results may be drawn from the concept of perceived quality among youngsters. The youngsters go and search for any news which they might think could be the attention seeking for their social network members and friends. They don’t bother whether this news is of good quality or not. Although, in different contexts, the perceived quality of news has been studied before. Due to modernization, viewers’ perceptions of quality and the objectives for which they obtain news are likely to have shifted. Quality perceptions within the reporting industry as well as from the perspective of users are not always in sync (Edgerly, 2021).

Rather than viewing news as something that “should be known” and using quality standards such as diversity or relevance, young viewers view news as something that is helpful, engaging, and entertaining to learn about (Galan et al., 2019). Although the direct relationship of news overload with news curation indicated that if there is a burden or overload of news among the readers and viewers, then it affects their behavior of news curation. Because if there is a lot of news available from multiple sources then social media users will have to do enormous news curation. The quantity of information is not always seen negatively because it presents information searchers with more options (Lee and Hong, 2021). However, the amount of information is enhanced since many news organizations republish the same or slightly modified versions of the very same news (Mourão and Robertson, 2019). People who sense overload but wish to continue viewing news items must control their overload in this case (Tunney et al., 2021).

This study also evaluated the mediating role of news avoidance between news relevance and curation, perceived news quality and news curation, and news overload and news curation. The indirect relationship of news avoidance between news relevance and news curation did not make any significance between these. This result indicated that direct relationship of news relevance with news curation could not be enhanced through news avoidance because news avoidance did not lead to news curation at any level. The effects could not be more enhanced. The other indirect relationship of news avoidance between perceived news quality and news curation indicated that this kind of direct relationship needed the help of news avoidance. This is due to the fact that news perceived quality could not make it to the social media users for news curation. For news curation, the users got help from news avoidance. It helped them for news curation regardless of the perceived quality of news. In a media world in which a wealth of news is available for 24 h a day, it is a contradiction that an increasing number of people are turning their backs on the news, resulting in widening inequities in current affairs awareness and involvement (Van Aelst et al., 2017).

Conversely, some new research of information dissemination have discovered a large population of individuals who read and watch no or just a little bit of news, referring them as minimalists, news avoiders, intermittent, or non-users (Wolfsfeld et al., 2015; Mosca and Quaranta, 2016; Strömbäck et al., 2018). Similarly the last indirect relationship referring mediating role of news avoidance between news overload and news curation could not find any significant contribution toward news curation. This analogy suggested that news sharing participants on social media did not bother about news overload and simply shared news regardless of its content, quality, relevance, and hefty amount. News avoidance could not aid this relationship, which was otherwise significant in this research. A plenty of research has undergone in past to examine different roles of different factors contributing toward news avoidance such as quality of news, relevance of news, efficacy of news, and new overload, etc., but no prior research has ever found mediating role of news avoidance in any context. This research would be a significant contribution in the field of news management.

### Theoretical Contribution

The present study contributes to the literature of journalism and online social media by measuring the impact of news relevance, perceived quality, and news overload on news curation. The study has contributed by finding that relevance of news has a negative significant impact on people’s news curation behavior, with people happily curating the news that they do not find relevant. Furthermore, this study gives evidence about the people curating the news they get if they are overloaded with the same topic and also so much news at once. They do not find the news interesting anymore and hence try sorting out the news according to their preference. Similarly, this study has greatly contributed to the literature by determining that news avoidance has a significant mediating role between the relationship of news quality and news curation.

### Limitations and Future Recommendations

Despite theoretical contribution to the literature, there are certain limitations associated with the present study. First of all, this study has considered only one dependent variable, i.e., news curation. In future more variables like news acceptance need for news and media literacy can be included in the framework to get a better insight in the news inclinations of the people. Furthermore, the present study has incorporated the mediating variable of media avoidance between the independent variables (news relevance, perceived quality, and news overload) and news curation. However, future studies can introduce more interesting variables like news seeking behavior, media literacy, etc. Moreover, the present study has been conducted in the Chinese context that should be replicated in other contexts to ensure the generalizability of the present conceptual framework.

### Practical Implications

The current study offers some valuable practical implications for the real world. First of all, this study gives an imminent implication to the news agencies in considering the relevance of the news according to the segment they are targeting. The news agencies and the social media platforms should avoid bombarding the public of general interest with the news that do not gain any attention from them. The target segments should be targeted subjectively rather than objectively. Furthermore, the study implies that the people are more prone to the quality of news rather than quantity; therefore, it is important for the news agencies to make sure that they produce and deliver the quality-based news considering the accuracy to penetrate the market at mass level. Furthermore, news overloading should be avoided because it reduces the ability to reach the people because during this process even the quality news get filtered, and people start avoiding the publishing organization or their online portal. Therefore, the news agencies must consider the relevance and quality of the news in order to avoid mass avoidance and filtration.

## Conclusion

In this post-COVID situation, seeking news and information has become an important part of daily life as it allows people to stay up to date with the recent news, allowing them to opt the best of available options. However, in this regard, relevance and perceived quality of news are very important to penetrate the masses such that if people are continuously provided with tons of news without maintaining the quality it would compel people to avoid them. Therefore, the present study has focused on examining the effect of news relevance, perceived quality, and news overload on the news curation with mediating role of news avoidance. The results of the study showed that news relevance and news overload have a negative significant effect on news curation. This shows that higher the relevance of news less will be the news curation. However, news overload showed a positive significant impact indicating that higher the news overload higher will be the news curation. Furthermore, the mediating role of news avoidance was found valid and significant between perceived quality and news curation.

## Data Availability Statement

The original contributions presented in the study are included in the article/supplementary material, further inquiries can be directed to the corresponding author.

## Author Contributions

All authors listed have made a substantial, direct, and intellectual contribution to the work and approved it for publication.

## Conflict of Interest

The authors declare that the research was conducted in the absence of any commercial or financial relationships that could be construed as a potential conflict of interest.

## Publisher’s Note

All claims expressed in this article are solely those of the authors and do not necessarily represent those of their affiliated organizations, or those of the publisher, the editors and the reviewers. Any product that may be evaluated in this article, or claim that may be made by its manufacturer, is not guaranteed or endorsed by the publisher.
